# Flash Communication: Mechanochemical Synthesis of Magnesium Anthracenes

**DOI:** 10.1021/acs.organomet.6c00107

**Published:** 2026-06-25

**Authors:** Dawid J. Babula, Nicholas J. Evans, Ross A. Jackson, Nicolas E. Mortreuil, Lewis G. Parker, Matthew P. Stevens, David J. Liptrot

**Affiliations:** † Department of Chemistry, University of Bath, Bath, BA2 7AY, U.K.; ‡ Institute of Sustainability and Climate Change, University of Bath, Bath, BA2 7AY, U.K.

## Abstract

Reaction of unactivated magnesium turnings, anthracene derivatives, and tetrahydrofuran for 1 h in a mixer mill (30 Hz) under argon provided convenient access to four magnesium anthracenes as their tris-THF complexes. Magnesium anthracene·THF_3_, magnesium 9-methylanthracene·THF_3_, magnesium 9,10-dimethylanthracene·THF_3_, and magnesium 9-phenylanthracene·THF_3_ were synthesized in good yields (83% (±1.2%), 85% (±0.5%), 85% (±0.8), and 87% (±1.1%), respectively). Analysis of these species by IR and NMR spectroscopy shows data consistent with material synthesized in solution.

## Graphical abstract

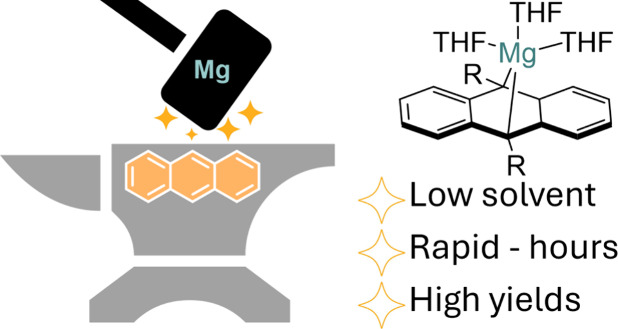

The magnesium-anthracene complex [Mg­(anthracene)­(THF)_3_] (**1**, THF = tetrahydrofuran) is an arene-stabilized, strongly reducing organomagnesium species whose solubility and reactivity can be tuned via variation in the ancillary donor ligand at magnesium or the substituents on the anthracene moiety.^1–6^ Its characteristic reactivity arises from the redox-active anthracene ligand, which can act as an electron reservoir, enabling coupled electron transfer and magnesium delivery to a substrate during reduction, often accompanied by the reformation of neutral anthracene upon reaction completion.^3,6–9^ This combination of high reducing power and a source of soluble, transferable magnesium, makes this class of complexes valuable in reductive magnesium reactivity and a useful reactant in both organic and inorganic reactions.^1,3,4,6^ ^,^ ^8,10–20^ Despite the utility of magnesium anthracenes, their wider use has been limited due to time-intensive preparations, which commonly require prolonged heating in donor solvents along with magnesium metal activators such as methyl iodide to overcome magnesium passivation.^6,21^ For example, Ramsden’s original report included a ca. 20 h reaction time,^21^ while Bogdanovic reported activation of the magnesium over 16 h followed by reaction over the course of 2 days.^22^

Mechanochemistry has emerged as a promising alternative approach for accessing organometallic compounds, including organomagnesium species, by enabling solid–solid contact while simultaneously generating a fresh reactive metal surface.^23–33^ In their seminal report, Ito and Kubota demonstrated that ball milling Mg(0) with organohalides in air affords the corresponding Grignard reagents in high yields with reaction times of approximately 1 h.^29^ Importantly, this method provided access to Grignard reagents from poorly soluble aryl halides that were previously inaccessible with conventional solution-based Grignard protocols.^29^ With this motivation, we set out to develop a mechanochemical protocol to access magnesium anthracene and its derivatives, with the aim of reducing reaction times, simplifying the operational procedure and broadening the accessibility of this reagent.

To investigate whether magnesium anthracene can be accessed under mechanochemical conditions, the synthesis of the parent [Mg­(anthracene)­(THF)_3_] was first explored. Reactions were conducted in the Retsch MM 400 Mixer Mill operating at 30 Hz, using four 5 mL steel jars with each one being loaded with a single Ø 7 mm steel ball. After screening different conditions and reaction times, the following optimized procedure was identified (Scheme 1); under an inert atmosphere in a glovebox, each steel jar was loaded with anthracene, unactivated magnesium turnings, and 9 equiv of THF. The reactants were milled for 1 h at 30 Hz, after which an orange slurry was obtained which is consistent with [Mg­(anthracene)­(THF)_3_] formation. This orange slurry was extracted from the four milling jars and volatiles were removed, leaving behind a free-flowing orange powder. This powder was subsequently washed with THF and dried in vacuo. The resulting product was confirmed to be [Mg­(anthracene)­(THF)_3_] by IR and ^13^C ssNMR spectroscopy which was in agreement with previously reported data. Working each of the jars up individually afforded isolated yields of 83% (±1.24%) of compound showing the consistency of this approach.^2,34^ Operational simplicity was increased by combining the four jars into a single flask work the workup stage (see ESI). The resultant material shows good stability in the glovebox, showing no evidence of decomposition over the course of weeks. This mechanochemical protocol is an improvement from previous solution-phase methods of accessing [Mg­(anthracene)­(THF)_3_], which typically take multiple days of stirring at room temperature, giving similar or lower yields.^6,22,35,36^

**Scheme 1:**
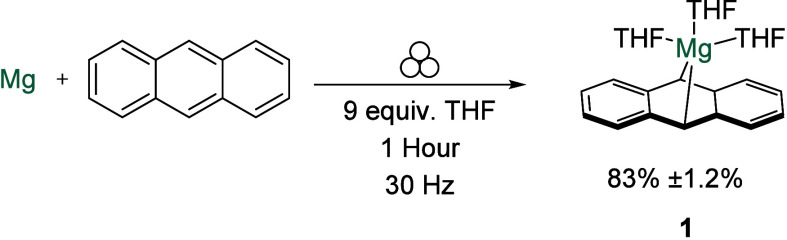
Mechanochemical Preparation of [Mg­(anthracene)­(THF)_3_]

With these optimized conditions, the application of this protocol was extended to 3 substituted anthracenes (Figure 1); 9-methylanthracene, 9,10-dimethylanthracene, and 9-phenylanthracene. Application of the same mechanochemical protocol as for [Mg­(anthracene)­(THF)_3_] afforded the corresponding substituted magnesium anthracene THF adducts (**2–4**) as orange, air-sensitive solids. In each case, after milling for 1 h, the corresponding magnesium anthracene complexes were obtained in yields of 85% (±0.5) (**2**), 85% (±0.8) (**3**), and 87% (±1.1%) (**4**). Across this series, the mechanochemical route to these magnesium anthracene complexes consistently improved throughput relative to solution-based methods, combining short reaction times with good-to-excellent isolated yields. Bogdanović and co-workers previously measured the solution-state stability of magnesium anthracenes, noting that at elevated temperature, magnesium anthracene derivatives revert to their constituent parent anthracenes and magnesium metal in THF solution.^12^ These high yields are, therefore, remarkable as no attempts to control heating of the reaction mixture due to friction, or exothermic reactions were made. These results further reinforce the advantages of low-solvent mechanochemical approaches, which can disfavor such deleterious back reactions.

**Figure 1:**
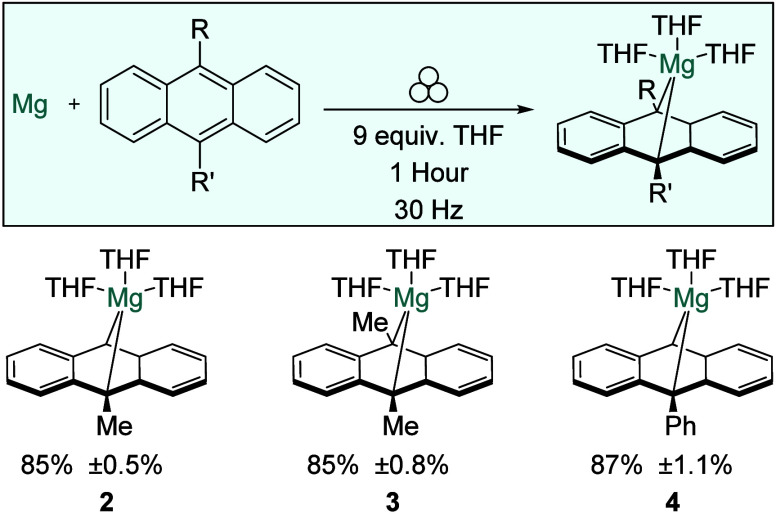
Substituted magnesium anthracene THF adducts obtained via mechanochemistry (isolated yields).

The identities of compounds **2–4** were confirmed by their IR and ^1^H NMR spectra, and the identities of compounds **2** and **3** were further confirmed by ^13^C ssNMR spectroscopy.^34,35^ The ^13^C ssNMR spectrum of **4** had not previously been reported, and we investigated this by ^13^C CP/MAS NMR spectroscopy using the TOSS technique. While the spectroscopic data associated with the arene fragments, and THF coligands of this species are relatively uninformative, the resonances associated with the 9- and 10-positions of the anthracene ring were more distinguishing. The unsubstituted 10-position shows a chemical shift essentially commensurate with those of **1** and **2** (δ_10‑C_ (ppm): **1**, 59.6; **2**, 59.8; **4**, 59.4), whereas the 9-position is shifted significantly downfield to 75.0 ppm. Raston and co-workers correlated these data with the nature of the magnesium–carbon bond,^34^ with a higher chemical shift reflecting greater sp^2^ character and thus reduced covalency. This significant shift reflecting a more ionic interaction is consistent with the expected effects of a phenyl substituent on the stability of a carbanion at the 9-position.

In summary, we have developed a mechanochemical route to access the parent [Mg­(anthracene)­(THF)_3_] adduct, which gave an isolated yield of 83% (±1.2%) following 1 h of milling of unactivated magnesium metal, anthracene, and THF under an inert atmosphere. This protocol improves and simplifies the widely used solution-phase synthesis methods, which typically require prolonged reaction times and activation of magnesium metal. This mechanochemical protocol is also applicable to substituted anthracenes, affording the corresponding magnesium anthracene THF adducts on the same time scale and in consistently good yields. These results establish mechanochemistry as a practical route to magnesium anthracene complexes and should facilitate broader access to magnesium anthracene reagents for activated magnesium chemistry and reductive transformations.

## Supplementary Material



## Notes

Experimental data, IR and NMR spectra (PDF)
