# Supplementary material for: Flash Communication: Mechanochemical Synthesis of Magnesium Anthracenes
Source: Organometallics. 2026 Jun 25;45(13):1469–72. doi: 10.1021/acs.organomet.6c00107 (PMC13371053; doi:10.1021/acs.organomet.6c00107)
Supplement: Supplementary file 1 [file om6c00107_si_001.pdf]

Supporting Information for: Flash Communication: Mechanochemical Synthesis of Magnesium Anthracenes

*Dawid J. Babula,[a,b] Nicholas J. Evans,[a] Ross A. Jackson,[a] Nicolas E. Mortreuil,[a] Lewis G. Parker,[a] Matthew P. Stevens,[a] David J. Liptrot[a,]**

a) Department of Chemistry, University of Bath, Bath, UK b) Institute of Sustainability and Climate Change, University of Bath, Bath, UK

Contents

General Information S2

Synthesis of Complexes S2

General Procedure A S2

General Procedure B S2

Synthesis of [Mg(anthracene)(THF)$_3$] (1) S3

Synthesis of [Mg(9-methylanthracne)(THF)$_3$] (2) S3

Synthesis of [Mg(9,10-dimethylanthracne)(THF)$_3$] (3) S3

Synthesis of [Mg(9-phenylanthracne)(THF)$_3$] (4) S4

ssNMR Spectra S5

Figure S2. ^{13}C CP/MAS NMR (5 kHz) spectrum of [Mg(9-methylanthracene)(THF)$_3$] (2). S5

Figure S3. ^{13}C CP/MAS NMR (5 kHz) spectrum of [Mg(9,10-dimethyanthracene)(THF)$_3$] (3)... S6

Figure S4. ^{13}C CP/MAS NMR spectrum(5 kHz) of [Mg(9-phenylanthracene)(THF)$_3$] (4). S6

IR Spectra S7

Figure S5. IR spectrum of [Mg(anthracene)(THF)$_3$] (1). S7

Figure S6. IR spectrum of [Mg(9-methylanthracene)(THF)$_3$] (2). S7

Figure S7. IR spectrum of [Mg(9,10-dimethylanthracene)(THF)$_3$] (3). S8

Figure S8. IR spectrum of [Mg(9-phenylanthracene)(THF)$_3$] (4). S8

^{1}H NMR Spectra S10

Figure S9. ^{1}H NMR spectrum (400 MHz, THF solvent suppression) of [Mg(anthracene)(THF)$_3$]. S10

Figure S10. ^{1}H NMR spectrum (400 MHz, THF solvent suppression) of [Mg(9-methylanthracene)(THF)$_3$]. S10

Figure S11. ^{1}H NMR spectrum (400 MHz, THF solvent suppression) of [Mg(9,10-dimethylanthracene)(THF)$_3$]. S11

Figure S12. ^{1}H NMR spectrum (400 MHz, THF solvent suppression) of [Mg(9-phenylanthracene)(THF)$_3$]. S11

References S12

General Information

All ball-milling experiments were set up in a glovebox and performed using a MM 400 Mixer Mill using 5 mL steel jars (Retsch brand, product 01.462.0290), each loaded with a single steel 7 mm Ø ball (2.1020 g, Retsch brand, product 05.368.0035) and sealed with electrical tape. Extractions, washings and dryings were carried out using Schlenk line techniques. Solid-state NMR spectra were recorded at 298 K on a Bruker 500 MHz spectrometer at 125.77 MHz (^{13}C) using a CP/MAS TOSS pulse sequence. All ^{1}H NMR spectra were collected on a Bruker Avance 400 with THF suppression. Anthracene and 9-methylanthracene were purchased from Fisher Scientific, 9,10-dimethylanthracene from Sigma-Aldrich, and 9-phenylanthracene from Fluorochem; all chemicals were used without further purification. Magnesium turnings were purchased from Sigma-Aldrich and used without further purification. THF was dried over sodium/benzophenone, distilled, and stored over molecular sieves.

Highly divided magnesium which can form during the milling reactions is extremely flammable, and may be pyrophoric. Its reaction with water is exothermic and releases hydrogen gas which may ignite. Care should be taken to meticulous exclude air, and to carefully quench reaction residues.

Synthesis of Complexes

General Procedure A

Four 5 ml steel jars, each equipped with a 7 mm Ø steel ball, were dried in an oven at 160°C and cycled into a glovebox over a period of 30 minutes. Each jar was charged with anthracene or one of its derivatives, along with magnesium turnings and 9 equivalents of THF. The jars were subsequently closed and sealed with electrical tape. All four jars were milled simultaneously for 1 hour at 30 Hz. The product was extracted from the jars with THF (3 x 1 mL), followed by removal of volatiles under reduced pressure. The resulting solid was washed with THF and dried *in vacuo*. A yield was collected for each jar

General Procedure B

General procedure A is followed up until the work-up stage. For practical reasons and to facilitate work-up, the reaction mixtures from the four jars were extracted with THF into one Schlenk, followed by the removal of volatiles, washing with THF (3 x 5 mL) and drying *in vacuo*. The yield was subsequently determined from the combined reaction mixtures.

Synthesis of [Mg(anthracene)(THF)$_3$] (1)

Following general procedure A, each steel jar was charged with anthracene (250 mg, 1.40 mmol), magnesium turnings (31.6 mg, 1.40 mmol) and 9 equivalents of THF (1.02 mL, 12.6 mmol) to access [Mg(anthracene)(THF)$_3$] as an orange solid with an average 83% (±1.24%) yield.

Jar 1: 0.4989 g, 84.9%

Jar 2: 0.4990 g, 84.9%

Jar 3: 0.4840 g, 82.4%

Jar 4: 0.4893 g, 83.3%

Average: 0.4928 g (±0.0072 g), 83.9 % (±1.24%)

Scaled Up Procedure

Following a modified general procedure B, eight 5 mL jars were used instead of four. Each jar was loaded with anthracene (458.3 mg, 2.571 mmol), Mg turnings (62.51 mg, 2.571 mmol) and 9 equivalents of THF (1.88 mL, 23.14 mmol). Milling and the work-up were carried out as described in the general procedure, giving access to [Mg(anthracene)(THF)$_3$] as an orange solid in 82% yield (7.0598 g, 16.855 mmol).

Synthesis of [Mg(9-methylanthracne)(THF)$_3$] (2)

Following the general synthesis procedure A, each steel jar was charged with 9-methyanthracene (250 mg, 1.30 mmol), magnesium turnings (31.6 mg, 1.30 mmol) and 9 equivalents of THF (949 μL, 11.7 mmol) to access [Mg(9-methylanthracne)(THF)$_3$] as dark orange solid with an average 85.2% (±0.52) yield.

Jar 1: 0.4760 g, 84.6%

Jar 2: 0.4830 g, 85.8%

Jar 3: 0.4811 g, 85.5%

Jar 4: 0.4784 g, 85.0%

Average: 0.4796 (±0.0031 g), 85.2 % (±0.52%)

Following the general synthesis procedure B, each steel jar was charged with 9-methyanthracene (250 mg, 1.30 mmol), magnesium turnings (31.6 mg, 1.30 mmol) and 9 equivalents of THF (949 μL, 11.7 mmol) to access [Mg(9-methylanthracne)(THF)$_3$] as dark orange solid in 88% yield (1.9807 g, 4.5755 mmol).

Synthesis of [Mg(9,10-dimethylanthracne)(THF)$_3$] (3)

Following the general synthesis procedure A, each steel jar was charged with 9,10-dimethyanthracene (250 mg, 1.21 mmol), magnesium turnings (29.5 mg, 1.21 mmol) and 9 equivalents of THF (885 μL, 10.9 mmol) to access [Mg(9,10-dimethylanthracene)(THF)$_3$] as dark orange solid with an average 85% (±0.82) yield.

Jar 1: 0.4658 g, 86 %

Jar 2: 0.4554 g, 84.1%

Jar 3: 0.4615 g, 85.2 %

Jar 4: 0.4580 g, 84.6 %

Average: 0.4602 g (±0.0045 g), 85 % (±0.82%)

Following the general synthesis procedure B, each steel jar was charged with 9,10-dimethyanthracene (250 mg, 1.21 mmol), magnesium turnings (29.5 mg, 1.21 mmol) and 9 equivalents of THF (885 µL, 10.9 mmol) to access [Mg(9,10-dimethylanthracene)$(THF)_3$] as dark orange solid in 86% yield (2.1664 g, 4.1955 mmol).

Synthesis of [Mg(9-phenylanthracne)$(THF)_3$] (4)

Following the general synthesis procedure A, each steel jar charged with 9-phenylanthracene (250 mg, 983 µmol), magnesium turnings (23.9 mg, 983 µmol) and 9 equivalents of THF (718 µL, 8.85 mmol) to access [Mg(9-phenylanthracne)$(THF)_3$] as dark orange solid with an average 87.58 % (±1.11%) yield.

Jar 1: 0.4280 g, 87.9 %

Jar 2: 0.4255 g, 87.46%

Jar 3: 0.4190 g, 86.12%

Jar 4: 0.4320 g, 88.79%

Average: Average Mass: 0.4261 (±0.0055 g), 87.58 % (±1.11%)

Following the general synthesis procedure B, each steel jar charged with 9-phenylanthracene (250 mg, 983 µmol), magnesium turnings (23.9 mg, 983 µmol) and 9 equivalents of THF (718 µL, 8.85 mmol) to access [Mg(9-phenylanthracne)$(THF)_3$] as dark orange solid in 89% yield (1.7405 g, 3.5164 mmol).

ssNMR Spectra

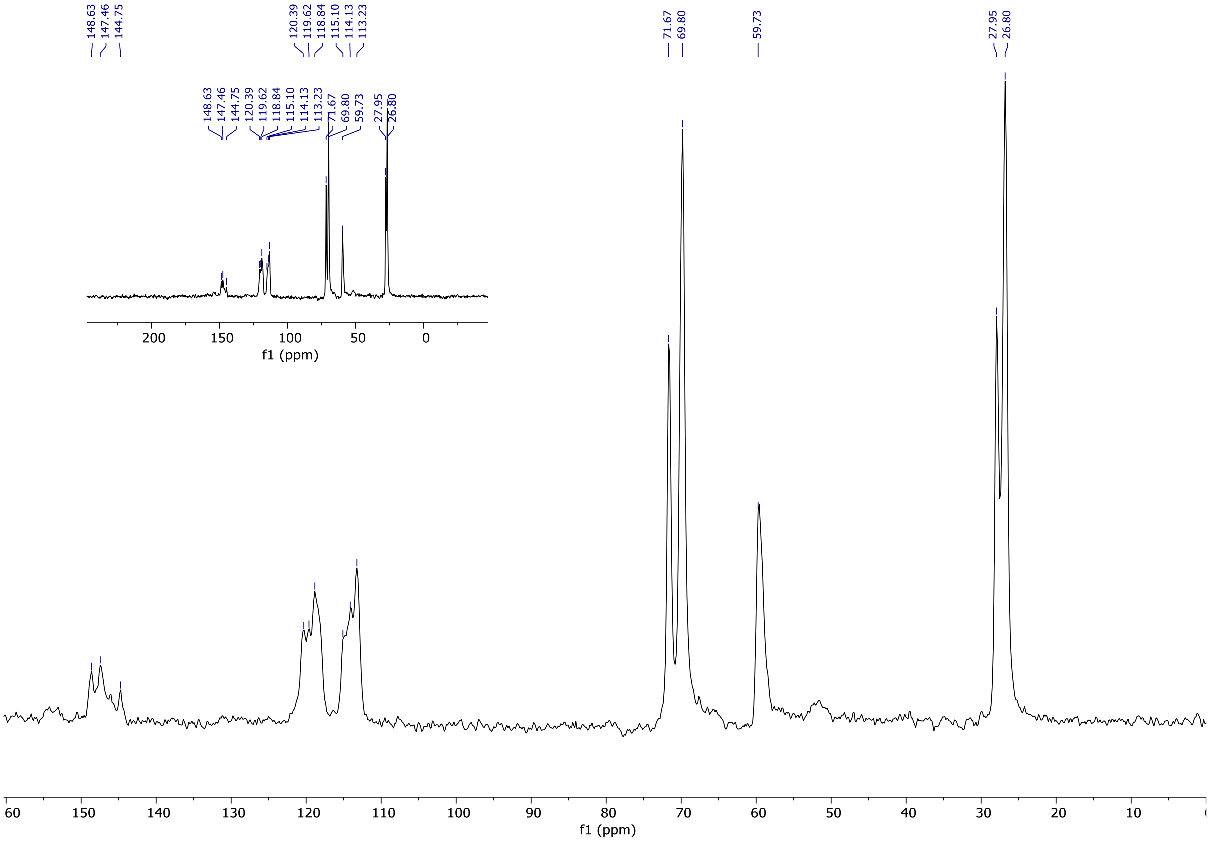

Figure S1. ^{13}C CP/MAS NMR (5 kHz) spectrum of [Mg(anthracene)(THF)$_3$].

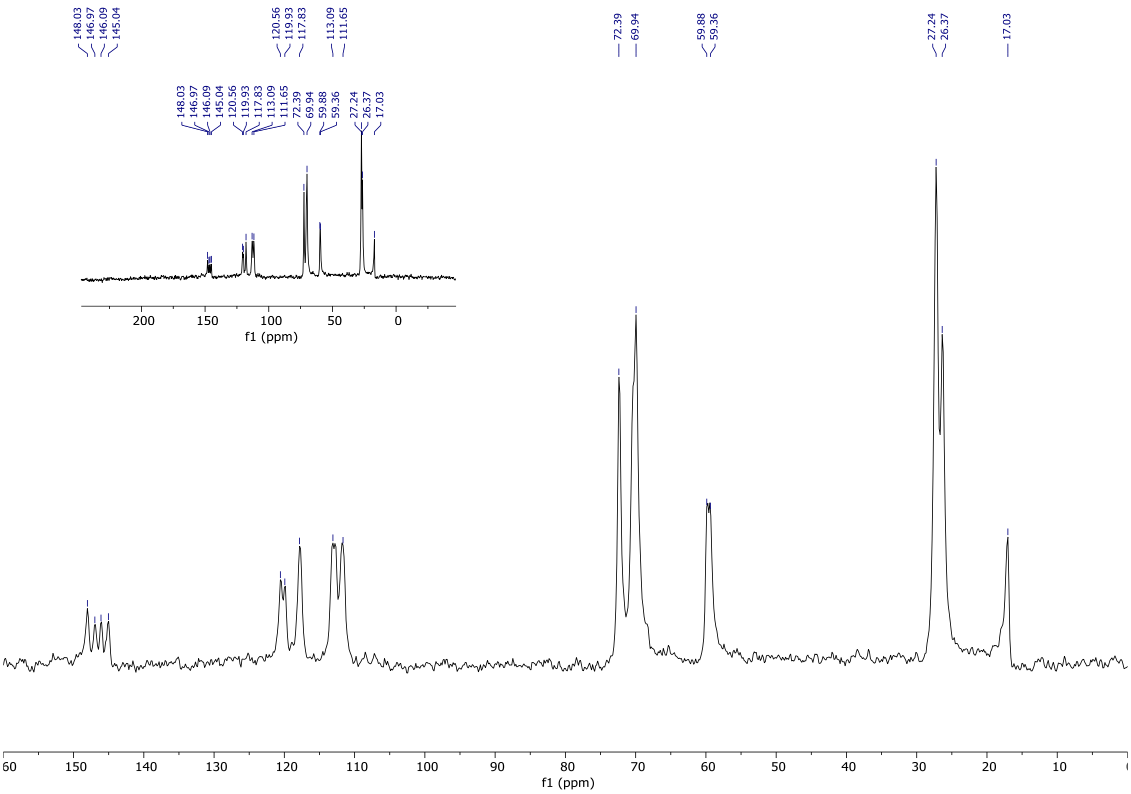

Figure S2. ^{13}C CP/MAS NMR (5 kHz) spectrum of [Mg(9-methylanthracene)(THF)$_3$].

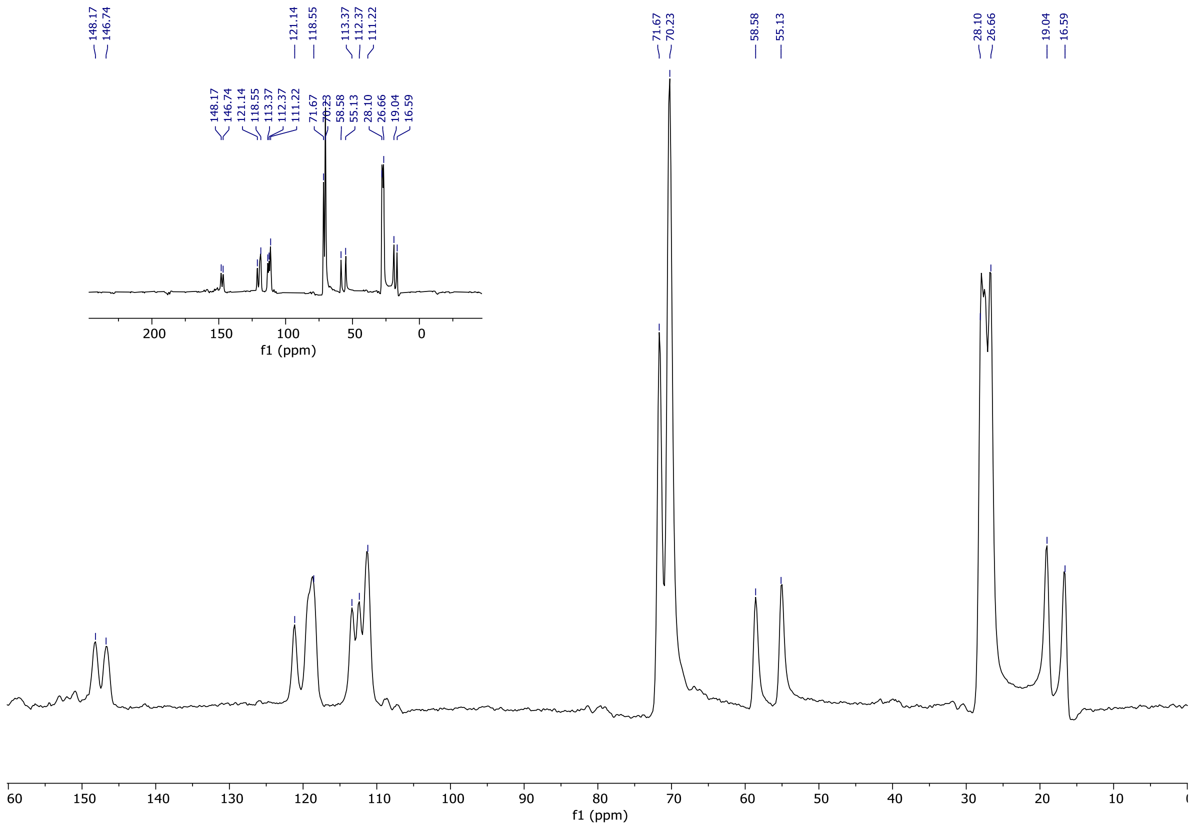

Figure S3. ^{13}C CP/MAS NMR (5 kHz) spectrum of [Mg(9,10-dimethyanthracene)(THF)$_3$].

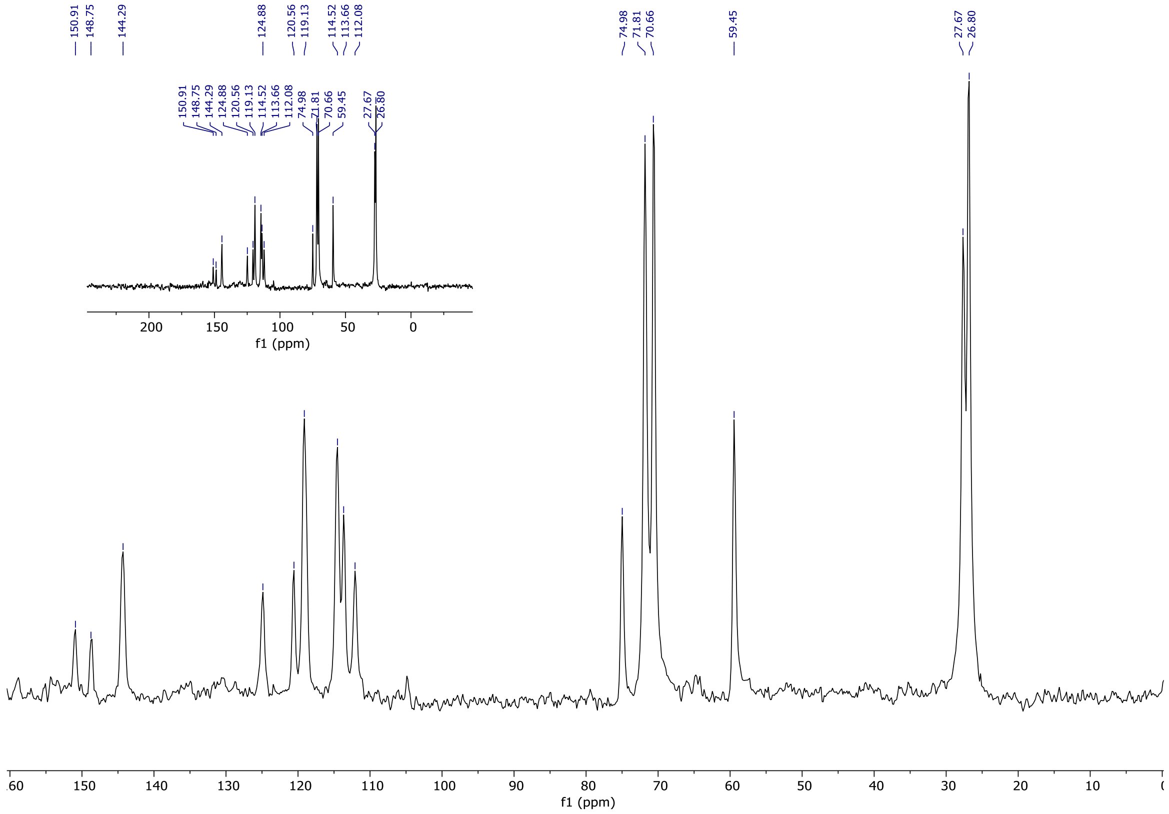

Figure S4. ^{13}C CP/MAS NMR spectrum(5 kHz) of [Mg(9-phenylanthracene)(THF)$_3$].

IR Spectra

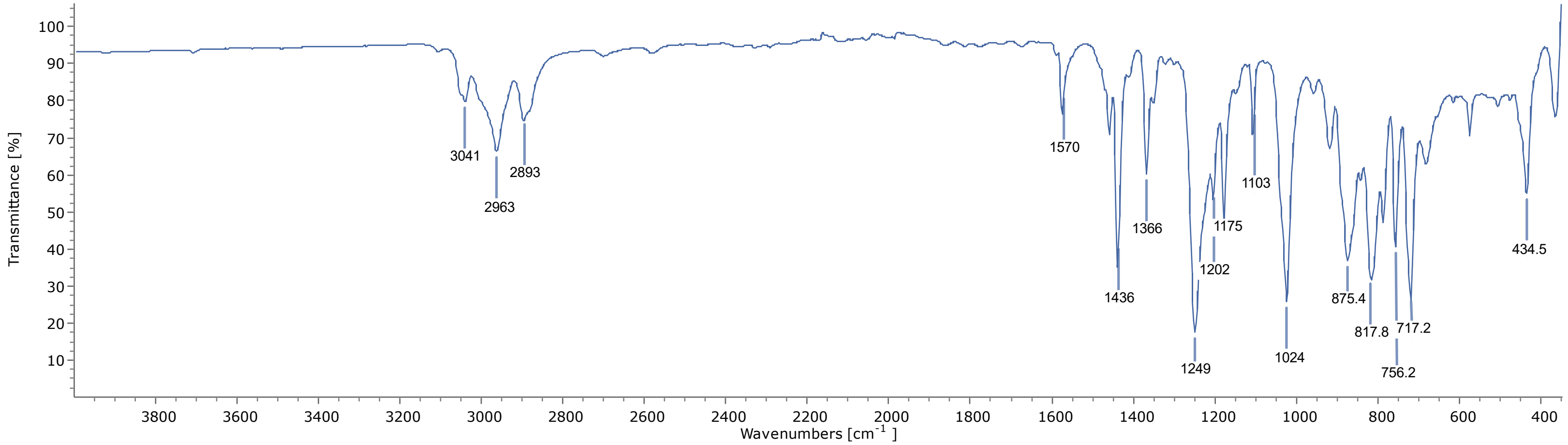

Figure S5. IR spectrum of [Mg(anthracene)(THF)$_3$].

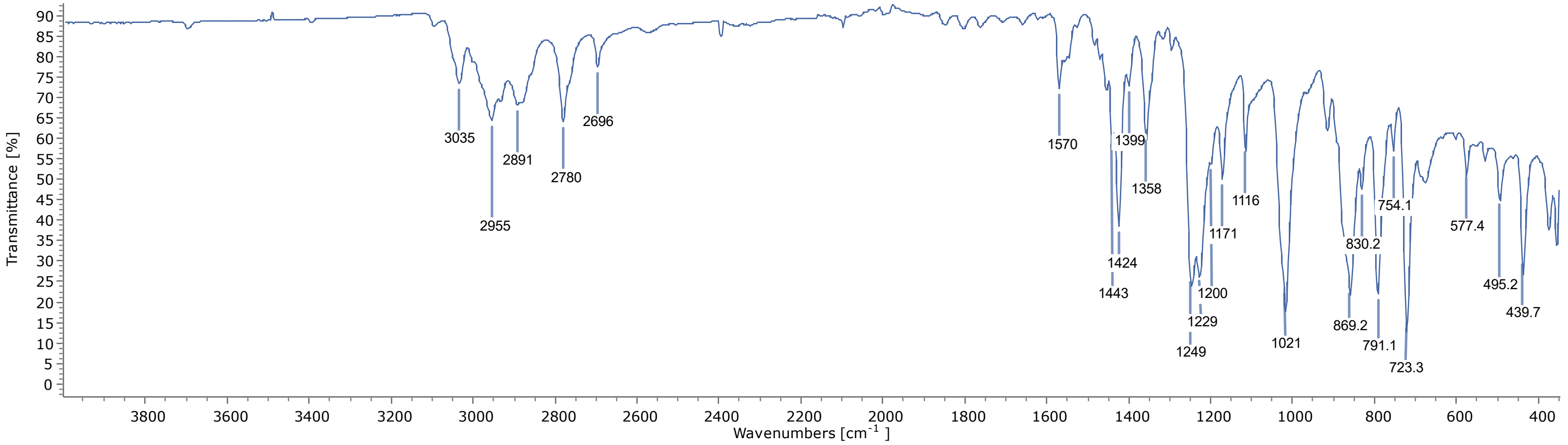

Figure S6. IR spectrum of [Mg(9-methylanthracene)(THF)$_3$].

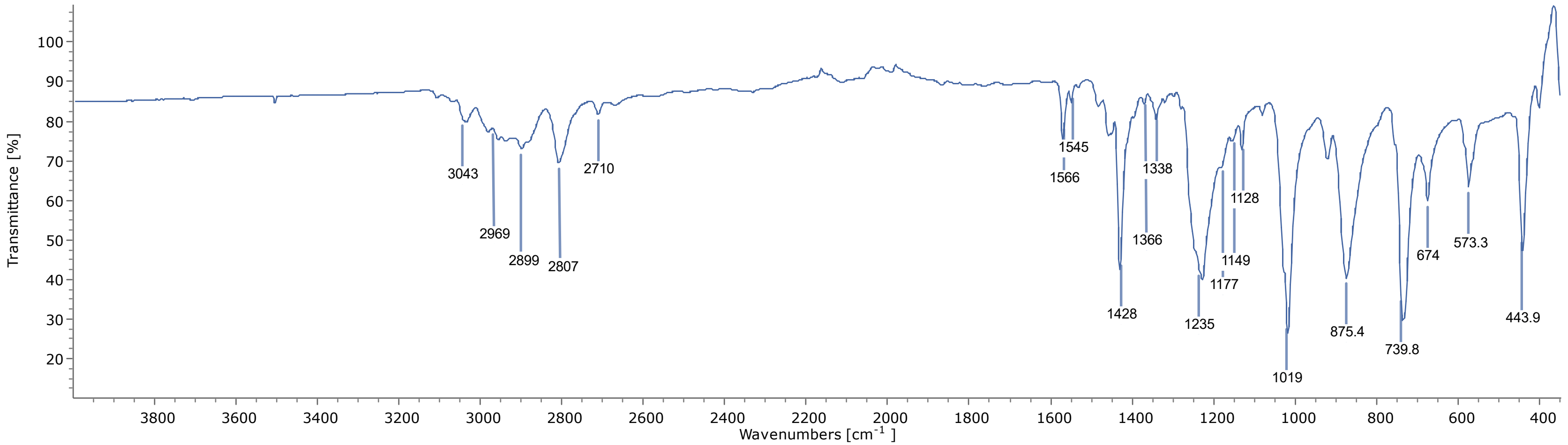

Figure S7. IR spectrum of [Mg(9,10-dimethylanthracene)(THF)$_3$].

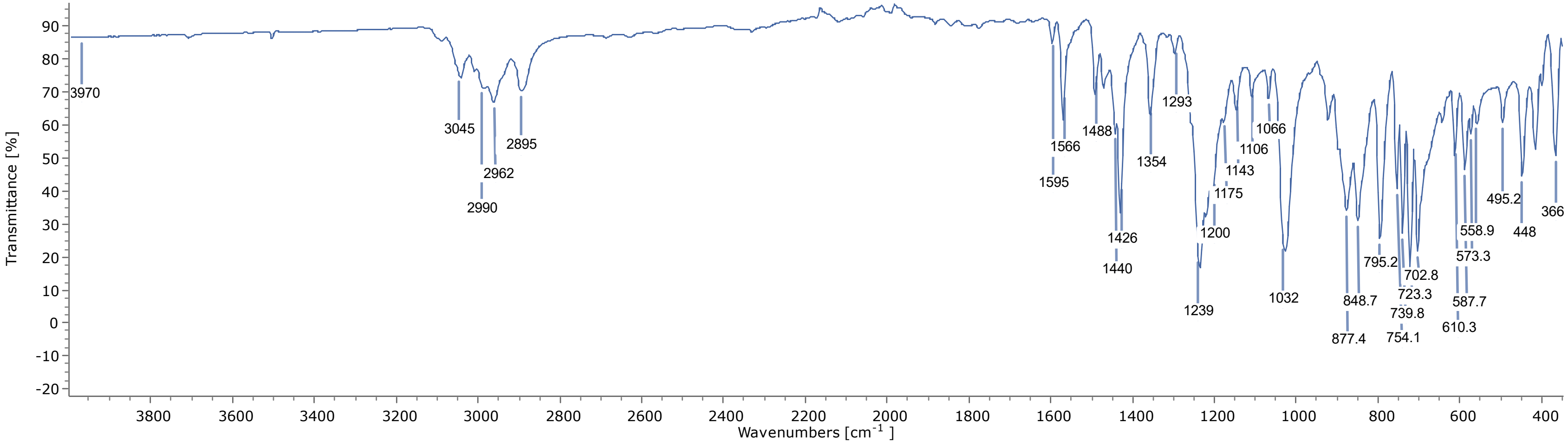

Figure S8. IR spectrum of [Mg(9-phenylanthracene)(THF)$_3$].

**[Mg(anthracene)(THF)$_3$]**		**[Mg(9-methylanthracene)(THF)$_3$]**		**[Mg(9,10-dimethylanthracene)]**		**[Mg(9-phenylanthracene)]**	
IR Stretching Frequency/ cm^{-1}							
This study	Reported[1]	This study	Reported[1]	This study	Reported[1]	This study	Reported[1]
434 m	434 m	439 s	439 s	443 m	443 m	448 m	448 m
717 s	717 s	495 m	496 m	573 m	537 m (*sic*)	495 w	495 w
756 m	756 m	577 w	577 w	674 m	673 w	558 w	558 w
817 s	817 s	723 s	724 s	739 s	739 s	573 w	572 w
875 s	875 s	754 w	754 w	875 s	876 s	587 m	588 s
1024 s	1024 s	791 s	790 s	1019 vs	~1022 vs	610 m	611 m
1103 w	1104 w	830 w	831 w	1128 w	1127 m	702 s	702 s
1175 w	1174 w	869 s	~870 s	1149 w	1149 w	723 s	723 s
1202 w	1201 w	1021 vs	1024 vs	1177 w	1177 w	739 s	739 s
1249 vs	1250 vs	1116 w	1115 m	1235 vs	~1235 vs	754 s	753 s
1366 m	1364 m	1171 m	1172 m	1338 m	1337 m	795 s	795 s
1436 s	1437 s	1200 w	1200 w	1366 w	~1365 w	848 m	849 m
1570 m	1570 m	1229 s	1229 s	1428 s	1428 s	877 m	876 vs
		1249 s	1251 vs	1545 w	1545 w	1032 vs	1031 vs
		1358 m	1358 m	1566 m	1565 s	1066 w	1064 m
		1399 w	1399 w			1106 m	1105 m
		1424 s	1425 s			1143 m	1142 m
		1443 m	1442 s			1175 w	1174 w
		1570 m	1570 m			1200 s	~1200 s
						1239 vs	~1240 vs
						1293 w	1292 m
						1354 m	1353 s
						1426 s	1425 s
						1440 s	1440 s
						1488 s	1488 s
						1566 s	1565 s
						1595 m	1592 m

Table S1. IR stretching frequencies of [Mg(anthracene)(THF)$_3$] (1), [Mg(9-methylanthracene)(THF)$_3$] (2), [Mg(9,10-dimethylanthracene)(THF)$_3$] (3), and [Mg(9-phenylanthracene)(THF)$_3$] (4) obtained in this study, compared with literature-reported values

^{1}H NMR Spectra

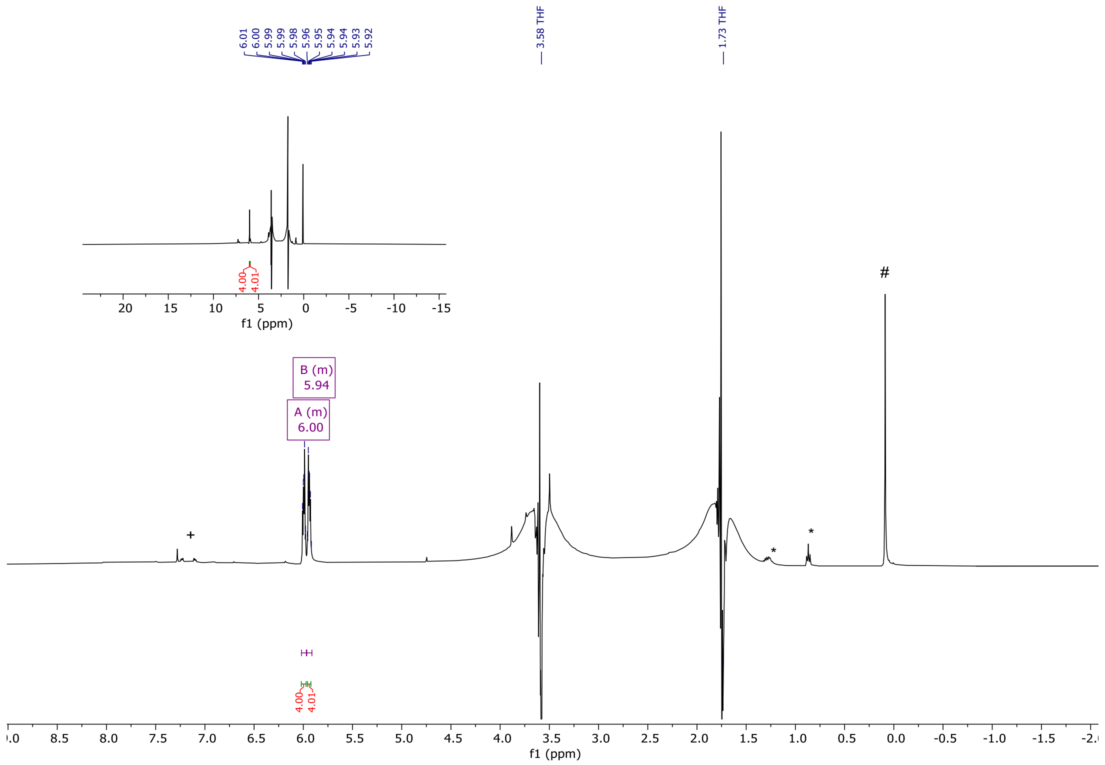

Figure S9. ^{1}H NMR spectrum (400 MHz, THF solvent suppression) of [Mg(anthracene)(THF)$_3$]. # - grease, * - pentane, + free anthracene

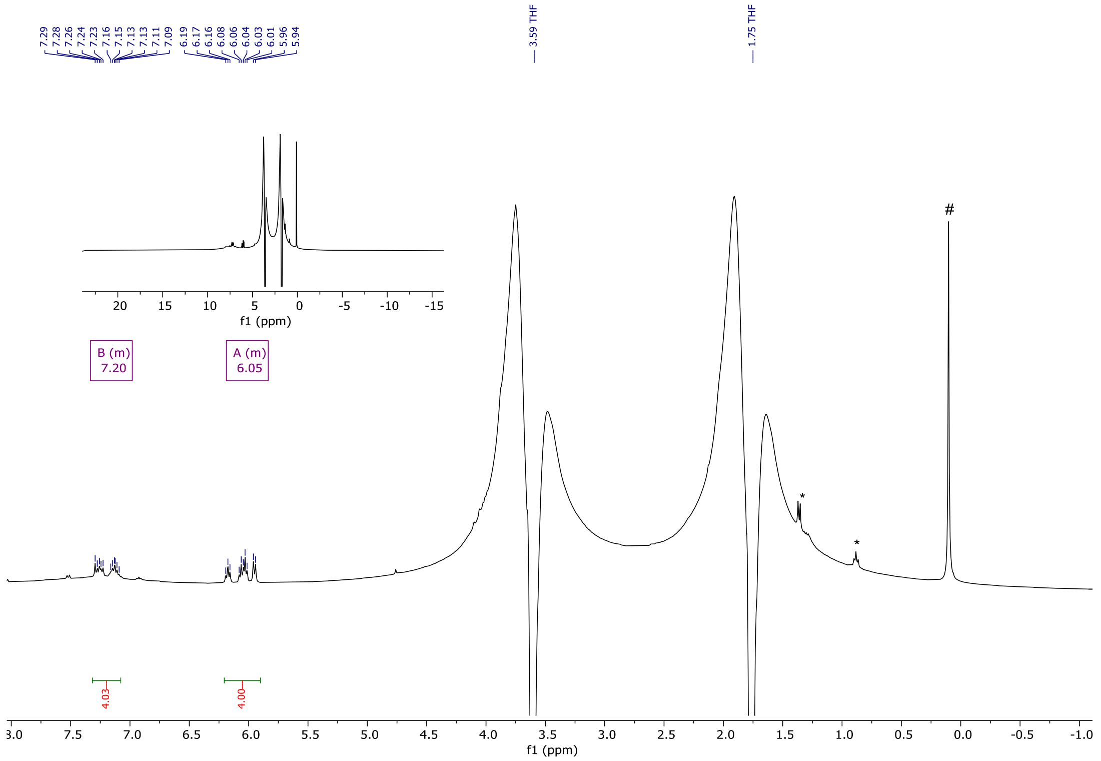

Figure S10. ^{1}H NMR spectrum (400 MHz, THF solvent suppression) of [Mg(9-methylanthracene)(THF)$_3$]. # - grease, * - pentane

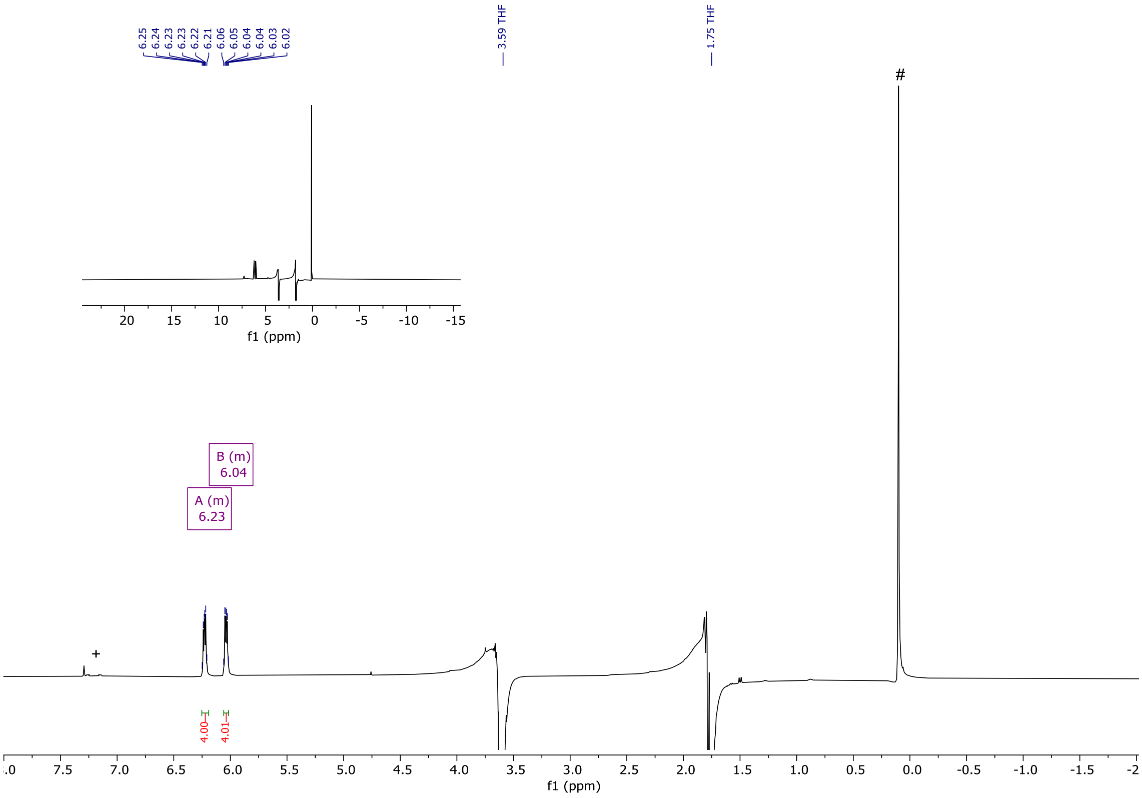

Figure S11. ^{1}H NMR spectrum (400 MHz, THF solvent suppression) of [Mg(9,10-dimethylanthracene)(THF)$_3$]. # - grease, * - pentane, + free anthracene

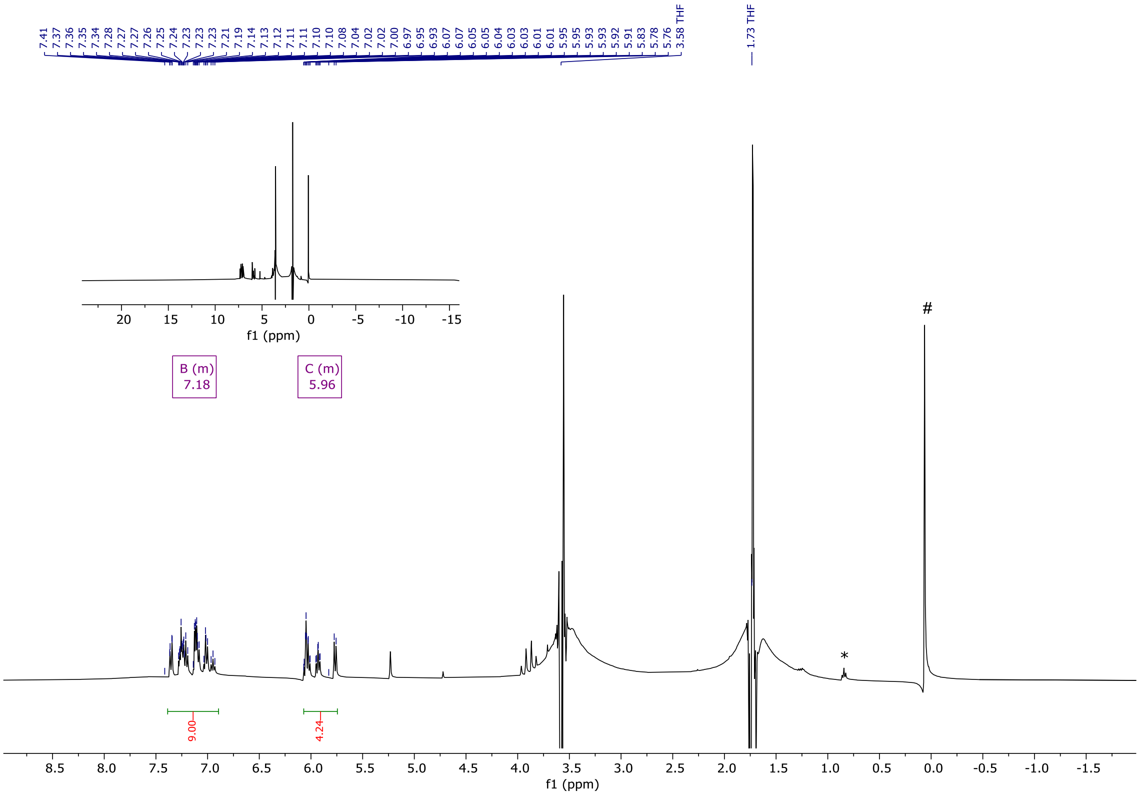

Figure S12. ^{1}H NMR spectrum (400 MHz, THF solvent suppression) of [Mg(9-phenylanthracene)(THF)$_3$]. # - grease, * - pentane

References

1. B. Bogdanovic̀, N. Janke, H.-G. Kinzelmann, K. Seevogel and J. Treber, *Chem. Ber.*, 1990, **123**, 1529-1536.
